# Evolution of the consumption trend of proton pump inhibitors in the Lleida Health Region between 2002 and 2015

**DOI:** 10.1186/s12889-022-13217-6

**Published:** 2022-04-24

**Authors:** F. Torres-Bondia, J. de Batlle, L. Galván, M. Buti, F. Barbé, G. Piñol-Ripoll

**Affiliations:** 1 Pharmacy Department, Clinical Neuroscience Research, IRB Lleida, Arnau de Vilanova University Hospital, Lleida, Spain; 2 Biomedical Research Networking Centre for Respiratory Diseases (Centro de Investigación Biomédica en Red de Enfermedades Respiratorias, CIBERES), Madrid, Spain; 3 Translational Research Group in Respiratory Medicine, Arnau de Vilanova University Hospital and Santa Maria University Hospital, IRB Lleida, Lleida, Spain; 4 Pharmacy Department, Servei Català de La Salut (Catalan Health Services), Lleida, Spain; 5 Unitat d’Avaluació Clínica (Clinical Evaluation Unit), Institut Català de La Salut (Catalan Institute of Health), Lleida, Spain; 6 Unitat Trastorns Cognitius (Cognitive Disorders Unit), Clinical Neuroscience Research, IRB Lleida, Santa Maria University Hospital, Rovira Roure nº 44, 25198 Lleida, Spain

**Keywords:** Proton pump inhibitors, Long term, Prescribing trends, Drug safety, Drug utilization

## Abstract

**Background:**

Proton pump inhibitors (PPIs) are one of the most commonly prescribed pharmacological groups. Their high prevalence and duration of use are of important health concern due to the risk they can cause to patients. Despite these risks, their use remains particularly high, especially in the elderly population. We determined the trend in the prevalence of PPI consumption in the population of the Lleida Health Region between 2002 and 2015 to explore patterns of use and associated characteristics.

**Methods:**

An analysis of secular trends between 2002 and 2015 was performed. The database included all individuals who used PPIs in the Lleida Health Region, which had 358.070 inhabitants in 2015. PPI use was evaluated using prescription dispensing data from the public health system. All types of PPIs approved by the pharmaceutical agency were included. Trends were investigated by age and sex.

**Results:**

For the whole study period, a total of 215,417 individuals accounted for 292,122 dispensations. Overall, 48% were women, and the mean age was 62 years. The dispensing prevalence of PPI use in 2015 was 18.0% overall—20.4% for women and 15.7% for men—and was 54.6% for those over 65 years. In terms of the subtypes of PPIs, 16.8% of prescriptions were for omeprazole, 0.66% were for pantoprazole, and 0.48% were for lansoprazole. The evolution of the annual PPIs dispensation prevalence showed a progressive increase from 11.3% in 2002 to 18.0% in 2015, which was attributable to an increase in the use of omeprazole (9.0% vs. 16.8%) and, to a lesser extent, esomeprazole (0.02% vs. 0.4%).

**Conclusion:**

An increase in the prevalence of PPI dispensation was observed over 14 years of follow-up. The prevalence of dispensation was especially high for the population older than 65 years, despite the risk of cognitive decline and falls. Comprehensive actions are required to to increase rational prescribing of PPIs, especially in high-risk populations.

**Supplementary Information:**

The online version contains supplementary material available at 10.1186/s12889-022-13217-6.

## Introduction

Proton pump inhibitors (PPIs) are among the most frequently prescribed pharmacological groups in both Europe and in the United States [1, 2]. In recent decades, the use of PPIs has increased; however, the prevalence of the conditions for which they are indicated (gastroesophageal reflux disease, nonerosive reflux disease, peptic ulcer disease and Zollinger-Ellison syndrome or the prevention of ulcers caused by nonsteroidal anti-inflammatory drugs) remains stable [3, 4]. Therefore, this growth is due in large part to their use for inappropriate indications. It is estimated that the PPIs are inappropriately used in approximately 50% of cases in both the hospital and outpatient setting [5], and this misuse is especially serious in the geriatric population, as different population studies have shown [6–10]. The most common inappropriate indications for which PPIs are used are gastroprotection in patients who are not taking drugs that are harmful to the gastric mucosa, prophylaxis for stress ulcers in low-risk patients and other related incorrect diagnoses [11]. In addition, the availability of generic PPI drugs has increased nonprescription use due to their low price, which has contributed to even higher consumption of these drugs [1].

Potential adverse effects of PPIs include community-acquired pneumonia, *Clostridium difficile* infections, osteoporosis and bone fractures, chronic kidney disease, vitamin B12 deficiency and increased risk of dementia, cancers and other malignant diseases with long-term use [12–14] (Table 1 Suppl Data).

In Spain, PPIs were the most prescribed pharmacological subgroup in terms of the number of packages provided by the National Health System in 2016. Approximately, one in 10 people takes a PPI daily. PPIs represent 7.4% of total packages and account for 3.4% of total national pharmaceutical spending [15]. Unlike studies of other pharmacological groups, such as BZD, that have been conducted in our country, there have been no specific campaigns aimed at reducing prescriptions for PPIs [16].

The objective of the present study was to determine the prevalence, patterns of use and characteristics associated with the use of PPIs in a population cohort in the Lleida Health Region (LHR) in Catalonia over a 14-year period between 2002 and 2015.

## Materials and methods

An analysis of prescription trends between January 1, 2002, and December 31, 2015 was performed. The database consisted of all individuals of any age and sex assigned to both physicians and basic health areas (a basic health area corresponds to the territory and population served by a primary care team comprising professionals in family medicine, paediatrics and nursing and administrative support personnel) of the LHR, which included 358,157 inhabitants in 2015.

To evaluate the consumption of PPIs, information provided by the public health system on the dispensation of these drugs by pharmacies was used. This information includes the number of containers dispensed. Spain has a public health system in which drugs are dispensed by pharmacies with a medical prescription (usually from a primary care physician or, sometimes, by a specialist). Distribution associated with mutual insurance companies or other insurers, medications administered to hospitalized patients, medications prescribed by private providers or medications dispensed without a prescription were excluded. In Spain, such cases represent less than 2% of all drug consumption.

The best data source for studies that evaluate the prescription and consumption of drugs is drug dispensing records because they are based on actual drug purchases. Both the external and internal validity of studies based on such data is high. Therefore, the use of current dispensing records allows a highly reliable analysis of drug consumption at the individual level [17, 18].

PPIs were categorized according to the Anatomic Therapeutic chemical (ATC) classification, as follows: A02BC01 (omeprazole), A02BC02 (pantoprazole), A02BC03 (lansoprazole), A02BC04 (rabeprazole) and A02BC05 (esomeprazole) [19]. All PPIs in the aforementioned groups that were listed as approved in the medicines catalogue of the Spanish Agency of Medicines during the study period were included [4]. The use of PPIs was defined as at least 1 prescription during the study period. Exposure to PPIs was based on the number of accumulated defined daily dose (DDDs) per individual during the study period. A DDD is defined as a technical unit of measurement that corresponds to the maintenance dose for the main indication for a given route of administration in adults. The DDDs of active ingredients are established by the World Health Organization (WHO) and are published on the website of the WHO Collaborating Centre for Drug Statistics Methodology [19].

Long-term consumption over the whole study period was defined as a DDD ≥ 180 DDD [20].

The following clinical and demographic variables were recorded: age, sex, type of basic health area (rural or urban) and diagnoses (hypertension, diabetes mellitus, hyperlipidaemia, myocardial infarction, stroke, Alzheimer's disease or other dementia, anxiety, insomnia and depressive syndromes) according to the International Classification of Diseases, 10th revision (2018), Clinical Modification (ICD-10-CM) [21].

### Statistical analyses

PPI consumption was based on absolute values and percentages or means and standard deviations. The prevalence of PPI use was calculated by age, sex and type of PPI among individuals of any age who filled at least 1 prescription for any PPI between January 1, 2002, and December 31, 2015. The prevalence of global dispensing was described for the entire study period, and the prevalence of annual dispensing was described for a given year. To calculate the percentages of the total LHR population, official figures for the region from the Statistical Institute of Catalonia (IDESCAT) were used. This research project, with code P16/109, was approved by the appropriate ethics committee (the Committee of Ethics and Clinical Research of Lleida (CEIC)).

A description of the study population was created based on absolute values and percentages or means and standard deviations. To calculate the percentages of the total population of the Health Region of Lleida, the official figures for that region were used. The dispensing prevalence of PPIs use was calculated by age, sex, and type of PPIs for individuals of any age who were charged for at least 1 prescription for any selected drug between January 1, 2002, and December 31, 2015. We considered global dispensing prevalence when we described the whole study period and annual dispensing prevalence when we described use over a given year.

## Results

During the period from 2002–2015, a total of 215,417 subjects in the LHR used PPIs. These individuals generated a total of 292,122 records of dispensed drugs that included the different types of PPIs. Table 1 shows the characteristics of the study population. In the final year of follow-up (2015), the mean age was 62 (21) years. Forty-eight percent of the consumers were male, and the majority of the subjects (61%) were assigned to a rural basic health area. Among the main pathologies of the study population were arterial hypertension (20.2%), dyslipidaemia (15.8%) and anxiety disorders (13.5%).

**Table 1:** Characteristics of consumers of Proton-pump inhibitors in the the study population between 2002 and 2015

Characteristic	n (%)
Sex: women	112,126 (52%)
Age categories	
< 16	867 (0%)
16–24	4553 (2%)
25–44	47,673 (22%)
45–64	64,802 (30%)
> 64	97,522 (45%)
Setting: rural	130,744 (61%)
Main diagnoses	
Alzheimer’s	1032 (0.5%)
Dementia	3375 (1.6%)
Depression	15,974 (7.4%)
Anxiety	29,151 (13.5%)
Sleep disorders	2707 (1.3%)
Affective disorders	3478 (1.6%)
Ischemic cardiomyopathy	6856 (3.2%)
Hypertension	43,465 (20.2%)
Diabetes	17,883 (8.3%)
Dyslipidaemia	34,081 (15.8%)
Other	100,554 (46.7%)

In this same year, 64,611 people obtained at least one PPI from the pharmacy, representing an annual dispensing prevalence of 18.04%. More women (20.4%) than men (15.7%) obtained PPIs. PPI use increased with age, reaching 54.6% in people over 65 years of age (Table 2). In terms of the type of PPI (Table 2), omeprazole was by far the most frequently dispensed PPI. Omeprazole had an annual dispensing prevalence of 16.8% in 2015, followed by pantoprazole (0.66%) and lansoprazole (0.48%). This prescription trend was observed for all age groups and both sexes.

**Table 2:** Proton-pump inhibitor dispensing prevalence in 2015 by sex and age (%)

	** < 16**	**16–24**	**25–44**	**45–64**	** > 65**	**Total**
**Men**						
Omeprazole	0.29	2.40	6.77	16.89	47.73	14.60
Pantoprazole	0.00	0.04	0.21	0.66	2.13	0.59
Lansoprazole	0.01	0.05	0.12	0.49	1.27	0.38
Rabeprazole	0.00	0.02	0.06	0.18	0.51	0.15
Esomeprazole	0.04	0.06	0.23	0.48	1.03	0.38
Total	0.32	2.53	7.19	18.23	51.36	15.69
**Women**						
Omeprazole	0.31	3.56	7.99	20.04	53.13	19.00
Pantoprazole	0.01	0.10	0.26	0.84	2.02	0.73
Lansoprazole	0.01	0.09	0.13	0.67	1.74	0.59
Rabeprazole	0.00	0.01	0.08	0.28	0.69	0.24
Esomeprazole	0.01	0.13	0.22	0.73	1.27	0.53
Total	0.33	3.78	8.42	21.73	57.18	20.43
**All**						
Omeprazole	0.30	2.96	7.35	18.40	50.78	16.78
Pantoprazole	0.01	0.07	0.23	0.75	2.06	0.66
Lansoprazole	0.01	0.07	0.13	0.58	1.54	0.48
Rabeprazole	0.00	0.02	0.07	0.23	0.61	0.20
Esomeprazole	0.03	0.09	0.23	0.60	1.17	0.45
Total	0.33	3.13	7.77	19.91	54.64	18.04

Long-term consumption of PPIs (cumulative DDD** ≥ **180) was 5% in subjects between 25 and 44 years old, 22% in those between 45 and 64 years old, and 94% in those over 65 years old (Table 2. Suppl Data). Data according cumulative DDD** > **365 are shown in Table 3 Suppl Data.

**Fig. 1:**
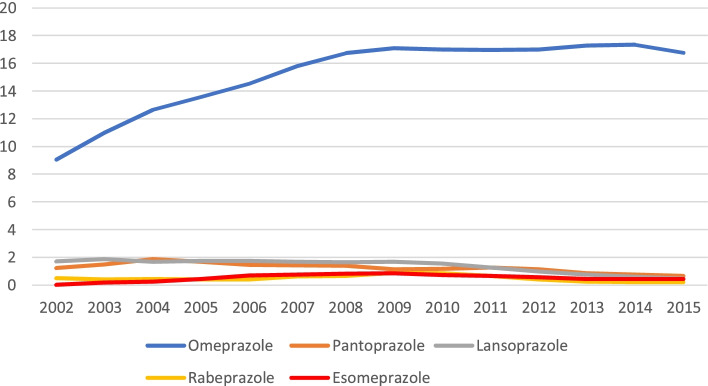
Proton-pump inhibitor dispensation prevalence by type from 2002 to 2015 (%)

**Fig. 2:**
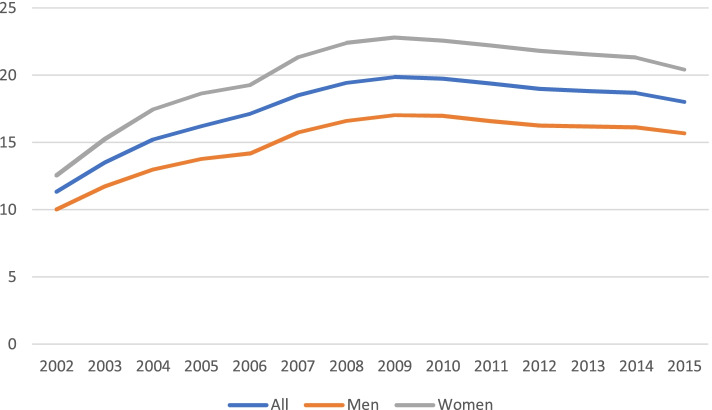
Proton-pump inhibitor dispensation prevalence by sex from 2002 to 2015 (%)

When we considered the evolution of the global dispensing prevalence over the study period, we observed a clear increase in the dispensation of PPIs, from 12.5% in 2002 to 18.1% in 2015 (Fig. 1). A significant increase was observed from 2002 to 2009, when the maximum annual dispensing prevalence of 21.6% was observed; starting that year, dispensation decreased slightly until 2015. No differences in the change in prescriptions in relation to sex were observed (Fig. 2).

**Fig. 3:**
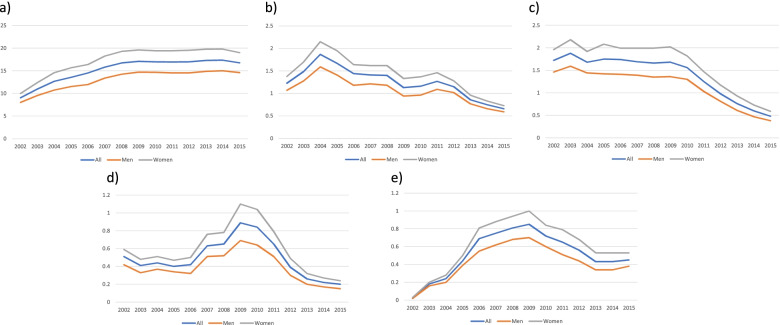
Proton-pump inhibitor dispensation prevalence by sex from 2002 to 2015 (%): **a**) omeprazole; **b**) pantoprazole; **c**) lansoprazole; **d**) rabeprazole; **e**) esomeprazole

When we analysed the evolution of use for the different types of PPIs, we observed a significant increase in the first years of follow-up for omeprazole (9.06% to 17.09% from 2002 to 2009), with a subsequent stabilization (16.98% to 16.78% of the 2010 to 2015). The increase from 2002–2015 was observed for both men and women, but the prevalence of use among women increased by 9% (from 10.02% to 19%), while use among men increased by 6% (from 8.02% to 14.6%) (Fig. 3a). Although the use of esomeprazole was much less prevalent than that of omeprazole, a decrease was also observed after 2009, but its use was much higher in 2015 (0.45%) than in 2002 (0.02%) (Fig. 3e).

Regarding the other PPIs, pantoprazole, lansoprazole and rabeprazole showed a clearly decreasing trend with slightly different evolutions over the study period (Fig. 3 b, c, d). With the exception of omeprazole and esomeprazole, the rest of the PPIs had a clearly lower dispensing prevalence in 2015 than in 2002.

**Fig. 4:**
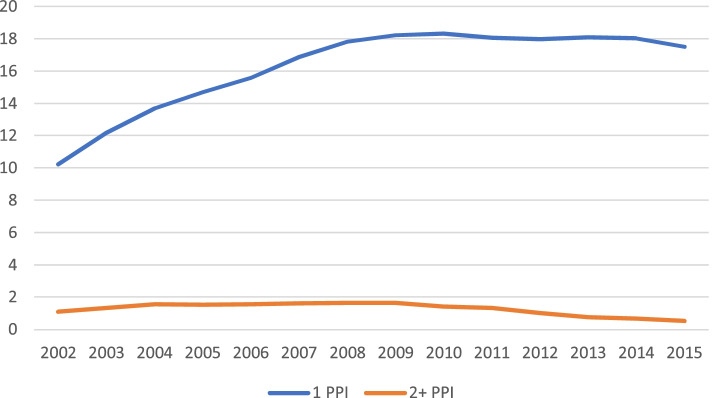
Dispensation prevalence of one or two or more proton-pump inhibitors from 2002 to 2015

When we considered the number of PPIs that the patients were taking, we found that in 2015, 0.51% of the population used two or more PPIs; this was a progressive decrease from 2002, when the prevalence was 1.1% (Fig. 4).

When we observed the prevalence of PPI use in relation to the use of other drugs, we observed throughout the study period, the subjects who used the most PPIs were those who did not use any other type of drug (7.53%), compared to the patients who consumed one (1.42%), two (2.72%) or three or more other drugs (6.37%). These data from 2015 were similar throughout the study period, with the patients who did not take any other drug and those that took more than three drugs showing the highest consumption of PPIs.

## Discussion

The results of the present study show a high prevalence of PPI use in a large population cohort throughout a 14-year observation period. Despite an insistence on the need to reduce the use of these medications, only a slight decrease in the consumption of some types of PPIs was observed in 2011; otherwise, there was a clear increase from 2002–2015, with a particularly high prevalence of use among the elderly population.

According to the latest report on the use of antiulcer drugs in Spain, from 2002 to 2012, the use of these drugs increased from 33.3 DHD (DDD/1000 inhabitants) in 2000 to 136.8 DHD in 2012, which represents an increase of 310.4%; this increase is partly explained by the increase in PPI use (> 500%). Among PPIs, the most commonly used was omeprazole, with a DHD of 18.1 DHD in 2000 and 104.0 in 2012. The use of other PPIs (esomeprazole, lansoprazole, pantoprazole and rabeprazole) also increased during this period, although to a lesser extent than omeprazole in absolute terms [22].

Our results are in line with those observed in different European and non-European countries [23, 24]. In France, where there are more studies on PPI use, the prevalence ranges between 19.5 and 33%. In general, PPI use seems to be higher in France than in other European countries, which report prevalences ranging from 7–18% [7, 25–27].

In contrast, in Denmark, the prevalence of PPI use increased by fourfold between 2002 and 2013, reaching 7.4% in 2014; however, even this peak prevalence is clearly lower than the prevalence observed in our study [28] and in other studies of similar populations, such as the Icelandic population, which also experienced an increase in PPI consumption between 2003 and 2015 (from 8.5 to 15.5%), although it was slightly lower than the increases observed in our study [29]. In Switzerland, an increase in PPI consumption from 19.7% to 23.0% was observed between 2012 and 2017, representing an increase of 4.8% vs. 6.4% [30].

Regarding population studies conducted in countries that are less comparable to ours, the prevalence of PPI use in the Australian population was 12.6% in 2016 [24], and it was 20–37% in hospitalized populations in China and Thailand [31, 32].

It stands out that the prevalence of consumption increased significantly with the age of the patients, reaching prevalences of 19.91% and 54.64% in individuals between 45 and 64 years and those older than 65 years, respectively. The Danish study also found that the prevalence increased significantly with age, reaching 20% in people over 80 years of age [28]. In the Australian study, the prevalence increased with age, especially after 65 years (33.4%), reaching 42.2% among people aged 75–84 years and 42.8% among people older than 85 years. This increase in the dispensation of PPIs with age was observed for both men and women [24] and was especially noticeable in those older than 75 years [26].

In terms of gender, we observed that the prevalence of PPI use was higher in women (20.43%) than in men (15.69%). Most of the articles in both European and non-European populations presented similar data [23, 28, 29], although in some, these differences were not observed [24, 30].

In general, the duration of treatment with PPIs that is recommended in clinical guidelines is 12 weeks [33]. Multiple definitions of long-term treatment are used in different studies [34]. Like some studies, such as the Australian study that defined long-term treatment as 3 months, we used a value of 180 DDD, which was based on 3 months of PPI use. In our study, we found that 25% of patients consumed more than 180 DDDs. This proportion was higher among elderly patients (93.9%) and lower in young people (< 25 years) (0.5%). This coincides with the fact that elderly adults are particularly vulnerable to polypharmacy and therefore are the population with the greatest need to avoid the prolonged use of PPIs [12]. Our results are similar to those of other studies, the majority of which found that PPIs were used both at higher doses than recommended and for longer durations, particularly in the elderly population [28, 30].

This excessive use of PPIs, often off-label, can be explained by the perception of PPIs as benign treatments with few adverse effects or because they are prescribed based on the clinical picture for patients (especially older patients) with symptoms suggestive of digestive pathology that require treatment but are not confirmed by endoscopy. It can also be explained by the increased used of antiplatelet drugs for primary prevention, which observational studies have shown increase the risk of bleeding [35, 36]. However, as different studies have shown, primary prophylaxis associated with the use of NSAIDs is often performed incorrectly in populations without risk factors for bleeding associated with NSAID use [27].

In our study, we did not have access to information regarding the reasons for PPI use or data regarding the prevalence of gastroesophageal reflux or peptic ulcer to allow a discussion of these factors.

### Limitations

This study has a number of limitations. The main one is the lack of data on the specific clinical indications for PPI use and whether PPIs were appropriately prescribed in the study population. Second, the prevalence data refer to the dispensation of the drugs by the public health system and not to their actual use. Although there are studies that have shown that the dispensation of drugs is well correlated with their use and offers better results than the use of prescription data, the limitations of using dispensation data should be considered [17]. Third, consumption was estimated using the DDD. The DDD values established by the WHO has additional limitations, since there may be differences between them and the actual doses used in clinical practice. However, this technical unit of measurement allows the comparison of consumption data among different countries. Fourth, the actual consumption of these drugs may have been higher than what was reflected in this study, since private dispensers and patients who took PPIs without a prescription were excluded. However, the denominator considered the population of the LHR, which was somewhat higher than the population that can obtain medications from the public health system. Finally, although the population included in the study was representative of the general population, it was not possible to ensure that the prescribing habits of family physicians in the LHR are representative of the prescribing habits of all family physicians in the nation.

## Conclusion

This study describes the trends in the consumption of PPIs over a 14-year period.

The use of these drugs increased significantly during the study period, despite showing a decrease in 2011, and remained especially high in the elderly population, which is more sensitive to the possible side effects of these medications.

While the consumption of pantoprazole, lansoprazole and rabeprazole decreased, the consumption of omeprazole and, to a lesser extent, esomeprazole increased significantly during the study period.

Since there are treatment alternatives with fewer side effects, and since other studies indicate that in many cases, these drugs are used off-label, especially for the elderly, efforts should be made to better educate doctors and patients to reduce the long-term inappropriate use of PPIs.

## Supplementary Information

**Additional file 1:** **Table 1**. Principal adverse effects of the different subtypes of PPIs. **Table 2**. Prevalence of long-term consumption of IBPs (cumulative DDD> 180 between 2002 and 2015), according to sex and age groups (%).**Table 3**. Prevalence of long-term consumption of IBPs (cumulative DDD> 365 between 2002 and 2015), according to sex and age groups (%).

## Data Availability

The datasets used and/or analysed during the current study are available from the corresponding author on reasonable request. Not repository is available.
