# Supplementary material for: Evolution of the consumption trend of proton pump inhibitors in the Lleida Health Region between 2002 and 2015
Source: BMC Public Health. 2022 Apr 24;22:818. doi: 10.1186/s12889-022-13217-6 (PMC9035259; doi:10.1186/s12889-022-13217-6)
Supplement: Supplementary file 1 — Additional file 1: Table 1. Principal adverse effects of the different subtypes of PPIs. Table 2. Prevalence of long-term consumption of IBPs (cumulative DDD> 180 between 2002 and 2015), according to sex and age groups (%).Table 3. Prevalence of long-term consumption of IBPs (cumulative DDD> 365 between 2002 and 2015), according to sex and age groups (%). [file 12889_2022_13217_MOESM1_ESM.docx]

**Evolution of the consumption trend of proton pump inhibitors in the Lleida Health Region between 2002 and 2015**

F Torres-Bondia^1*^, MD, PhD; J de Batlle^2,3*^, PhD; L Galván^4^, MD; M Buti^5^, MD; F Barbé^2,3^, MD, PhD; G Piñol-Ripoll^6^, MD, PhD

1. Pharmacy Department, Clinical Neuroscience Research, IRB Lleida, Arnau de Vilanova University Hospital, Lleida, Spain.
2. Biomedical Research Networking Centre for Respiratory Diseases (Centro de Investigación Biomédica en Red de Enfermedades Respiratorias, CIBERES), Madrid, Spain.
3. Translational Research Group in Respiratory Medicine, Arnau de Vilanova University Hospital and Santa Maria University Hospital, IRB Lleida, Lleida, Spain.
4. Pharmacy Department, Servei Català de la Salut (Catalan Health Services), Lleida, Spain.
5. Unitat d'Avaluació Clínica (Clinical Evaluation Unit), Institut Català de la Salut (Catalan Institute of Health), Lleida, Spain.
6. Unitat Trastorns Cognitius (Cognitive Disorders Unit), Clinical Neuroscience Research, IRB Lleida, Santa Maria University Hospital, Lleida (Spain).

* Co-first authors: FT-B and JdB contributed equally to this study.

Corresponding author:

Gerard Piñol Ripoll

Cognitive Disorders Unit

Hospital Santa Universitari Maria

Rovira Roure nº 44, 25198, Lleida, Spain

Phone: 34-937-727222. Ext.173. Fax: 34-976-727366

Email: gerard_437302@hotmail.com

**Table 1. Principal adverse effects of the different subtypes of PPIs.**

| Advers Events | PPI | | | | |
| --- | --- | --- | --- | --- | --- |
|  | **Omeprazole** | **Pantoprazole** | **Lansoprazole** | **Esomeprazole** | **Rabeprazole** |
| Abdominal pain | ✓ | ✓ | ✓ | ✓ | ✓ |
| Asthenia | ✓ | ✓ | ✓ | ✓ |  |
| Blurred vision | ✓ | ✓ | ✓ | ✓ | ✓ |
| Bone fracture | ✓ | ✓ | ✓ | ✓ | ✓ |
| *Clostridiodes difficile* associated diarrea | ✓ | ✓ | ✓ | ✓ | ✓ |
| Constipation | ✓ | ✓ | ✓ | ✓ | ✓ |
| Dementia | ✓ | ✓ | ✓ |  |  |
| Diarrhea | ✓ | ✓ | ✓ | ✓ | ✓ |
| Dry mouth | ✓ |  |  |  |  |
| Flatulence | ✓ | ✓ | ✓ | ✓ | ✓ |
| Gastric polyp | ✓ | ✓ | ✓ | ✓ | ✓ |
| Headache | ✓ | ✓ | ✓ | ✓ | ✓ |
| Hepatotoxicity | ✓ | ✓ | ✓ | ✓ | ✓ |
| Hypergastrinema | ✓ | ✓ | ✓ |  |  |
| Hypomagnesemia | ✓ | ✓ | ✓ | ✓ | ✓ |
| Nausea | ✓ | ✓ | ✓ | ✓ | ✓ |
| Renal disease | ✓ | ✓ | ✓ | ✓ | ✓ |
| Vomiting | ✓ | ✓ | ✓ | ✓ | ✓ |
| Vertigo | ✓ | ✓ | ✓ | ✓ | ✓ |
| Skin rash | ✓ | ✓ | ✓ | ✓ | ✓ |
| Upper respiratory infection | ✓ | ✓ | ✓ | ✓ | ✓ |
| Vitamin B12 deficiency | ✓ | ✓ |  | ✓ |  |

PPI: proton pump inhibitors (From Agencia Española de Medicamentos y Productos Sanitarios [https://www.aemps.gob.es/] Accessed 01 Feb 2022).

**Table 2. Prevalence of long-term consumption of IBPs (cumulative DDD> 180 between 2002 and 2015), according to sex and age groups (%).**

**Table 3. Prevalence of long-term consumption of IBPs (cumulative DDD> 365 between 2002 and 2015), according to sex and age groups (%).**
